# Metallopanstimulin-1 (MPS-1) mediates the promotion effect of leptin on colorectal cancer through activation of JNK/c-Jun signaling pathway

**DOI:** 10.1038/s41419-019-1911-8

**Published:** 2019-09-10

**Authors:** Dongxing Cao, Yang Luo, Shaolan Qin, Minhao Yu, Yifei Mu, Guangyao Ye, Nailin Yang, Zhijie Cong, Jianjun Chen, Jun Qin, Ran Cui, Ran Jing, Hui Cao, Ming Zhong

**Affiliations:** Department of Gastrointestinal Surgery, Ren Ji Hospital, School of Medicine, Shanghai Jiao Tong University, 200127 Shanghai, China

**Keywords:** Cancer, Mechanisms of disease

## Abstract

Obesity is a major epigenetic cause for colorectal cancer (CRC). Leptin is implicated in obesity-associated CRC, but the underlying mechanism remains unclear. The current study identified over-expression of metallopanstimulin-1 (MPS-1) in CRC patients through microarray and histological analysis, especially in obese CRC patients. MPS-1 was correlated with advanced tumor stage, suggesting its association with CRC progression. In addition, MPS-1 over-expression was associated with poor overall survival (OS) in obese CRC patients, but not in their non-obese counterparts, suggesting its potential as a prognostic marker of obese CRC patients. MPS-1 expression was positively associated with circulating leptin levels in CRC patients, especially in obese cases. Functional experiments demonstrated that MPS-1 silencing inhibited tumor proliferation and colony formation, and induced apoptosis of CRC cells in vitro. Converse results were obtained from the experiments with MPS-1 over-expression. Mechanistically, MPS-1 executed its action through induction of c-Jun N-terminal kinase (JNK)/c-Jun pathway. Moreover, the promotion effect of MPS-1 on CRC progression was modulated by leptin. In vivo studies demonstrated that MPS-1 silencing suppressed tumor growth of CRC via inhibiting JNK/c-Jun signaling. Collectively, this study indicates that MPS-1 promotes leptin-induced CRC via activating JNK/c-Jun pathway. MPS-1 might represent a potent candidate for the treatment and prognostic prediction of obesity-associated CRC.

## Introduction

Obesity is a global health challenge with a number of co-morbidities including enhanced risk of colorectal cancer (CRC)^1^. Among proposed mechanisms linking adiposity to CRC, much attention has been drawn to the deregulation of adipokine signaling, with a focus on leptin^2^. Leptin is an adipocyte-derived hormone that is up-regulated in obesity and plays a central role in regulating energy homeostasis^3^. Despite emerging evidences support leptin as a mediator of obesity-associated CRC^4^, the mechanisms underlying this pathobiology remain unclear.

Metallopanstimulin-1 (MPS-1), also known as ribosomal protein S27 (RPS27), is a multifunctional protein ubiquitously expressed in most normal human tissues^5^. MPS-1 plays a multifaceted role in different types of tumors. While MPS-1 was regarded as a tumor suppresser in head and neck squamous cell carcinoma^6^, it was found up-regulated in several forms of malignancies, such as breast cancer^7,8^ and gastric cancer^9–11^. For example, previous studies have revealed that knockdown of MPS-1 could inhibit growth and induce apoptosis of gastric cancer cells both in vitro and in vivo by suppressing NF-κB-signaling pathway^11^. So far, the implication of MPS-1 in CRC has rarely been reported except in a case report. In a patient with CRC, Ganger et al. observed that MPS-1 over-expression in tumor tissues and negative expression in the adjacent normal mucosa, and proposed a correlation between MPS-1 with a more aggressive behavior of CRC^12^. Nevertheless, results from large cohort studies and functional experiments are lacking.

Here, using microarray and histological analysis, we identified MPS-1 over-expression in CRC, especially in obese CRC patients. We evaluated the association between MPS-1 and prognosis and circulating leptin level of CRC patients, especially in the context of obesity. Functionally, the influences of MPS-1 on CRC progression and the underlying mechanism were investigated in vitro and in vivo. The crosstalk between MPS-1 and leptin was also assessed. These findings outline a role for MPS-1 in leptin-related CRC.

## Materials and methods

### Patients recruitment

The study included three independent patient cohorts at Department of Gastrointestinal Surgery, Ren Ji Hospital, School of Medicine, Shanghai Jiao Tong University. Cohort 1 included five CRC patients who received colectomy during January 2016–June 2018 for microarray analysis. Cohort 2 consisted of 40 CRC patients (20 obese and 20 non-obese cases) who received colectomy during January 2016–June 2018. Cohort 2 was included to verify the findings in microarray and to investigate the correlation between MPS-1 and serum leptin. Cohort 3 recruited 155 patients (83 obese and 72 non-obese cases) who underwent colectomy during January 2012–June 2014 for survival analysis. The patients who did not possess integrated follow-up information were excluded from cohort 3. All patients were confirmed CRC by pathological diagnosis. Tumor staging was performed according to American Joint Committee on Cancer (AJCC) 8th edition manual. Obesity was defined by body mass index (BMI) ≥ 30 kg/m^2^ according to the World Health Organization (WHO) classification^13^. Tumors and normal colon tissues were collected during operation. Normal colon tissues meant adjacent colonic mucosa more than 10 cm away from the tumor. The procedures were approved by the hospital Clinical Research Ethics Committee and informed consents were obtained.

### Microarray analysis

Total RNA was extracted from tissues by Trizol reagent (Invitrogen, Carlsbad, CA, USA). RNA quantity and quality were assessed with Thermo Nanodrop 2000 (Thermo Fisher Scientific, Wilmington, DE, USA) and Agilent 2100 Bioanalyzer (Agilent, Santa Clara, CA, USA). Affymetrix PrimeView Human Gene Expression Arrays (Thermo Fisher Scientific, Willmington, DE, USA) were applied for microarray analysis according to the manufacturer’s instruction. RNA was labeled by biotin using GeneChip 3′ IVT Express kit (Affymetrix). After hybridization overnight, the chips were stained by GeneChip™ Hybridization wash and stain kit (Affymetrix), and scanned with GeneChip Scanner 3000 according to the manufacturer’s protocol. Significantly differentially expressed genes were selected based on this threshold: FDR < 0.05 and absolute fold change >2.

### Cell culture

Human CRC cell lines RKO, DLD-1, HT29, SW480, HCT116, and Caco2, and normal colon epithelial cell FHC were purchased from BeNa Technology (Beijing, China). Cells were grown in six-well plates at 37 °C in a humidified atmosphere containing 5% CO_2_ and 95% air. SW480 cells were cultured using DMEM (Invitrogen, Carlsbad, CA, USA) supplemented with 10% FBS (Gibco, Rockville, MD, USA). RKO, HCT116, DLD-1, and FHC cells were cultured using RPMI1640 (Thermo Fisher Scientific, Wilmington, DE, USA) with 10% FBS. HT-29 was cultured with McCoy’s 5A medium (Thermo Fisher Scientific) with 10% FBS. CaCo_2_ was cultured with MEM (Thermo Fisher Scientific) supplemented with 20% FBS. Cell culture medium was changed every 72 h. To detect the impact of leptin, cells were cultured in the presence or absence of recombinant human leptin (50 ng/mL, # L4146, Sigma, Merck, Darmstadt, Germany) for 24 h. To detect the impact of anisomycin, cells were cultured in the presence or absence of anisomycin (0.01 μM, Beyotime, Shanghai, China) for 48 h.

### Real-time qPCR

Total RNA was extracted using TRIzol^®^ reagent (Thermo Fisher Scientific, Wilmington, DE, USA) according to the manufacturer’s protocol. The purity and integrity of RNA was assessed by Nanodrop 2000 spectrophotometry. Then, cDNA was generated by HiScript Q RT SuperMix for qPCR (+gDNA wiper) (Vazyme, Nanjing, Jiangsu, China) according to the manufacturer’s protocol. qPCR was performed using AceQ qPCR SYBR Green Master Mix (Vazyme). Gene expression was calculated using the comparative Ct method. GAPDH was used as the endogenous control. The primers for qPCR are listed in Table S1.

### Western blotting (WB)

CRC tissues and cells and FHC cells were lysed in ice-cold radio immunoprecipitation assay (RIPA) buffer (Millipore, Temecula, CA, USA) for 30 min. Total protein concentration was determined with BCA Protein Assay Kit (Thermo Fisher Scientific, Waltham, MA, USA). Protein samples (20 µg in each lane) were separated by 10% SDS–PAGE (Invitrogen, Carlsbad, CA, USA), and were subsequently transferred onto polyvinylidene fluoride (PVDF) membrane. Blots were incubated with 5% BSA in Tris-buffered saline containing 0.5% Tween 20 (TBST) for 60 min, and incubated overnight at 4 °C with corresponding primary antibodies. Membranes were then incubated for 1 h in appropriate secondary antibody. Proteins were visualized by enhanced chemiluminescence (ECL). Antibodies are listed in Table S2.

### Immunohistochemical staining (IHC)

Paraffin-embedded CRC and normal tissue samples from CRC patients were cut into 4 μm sections. The samples were blocked and then incubated with anti-MPS-1, anti-p-JNK (c-Jun N-terminal kinase), anti-p-c-Jun, and anti-Ki-67 at 4 °C overnight in incubator. Next, the sections were incubated with goat anti-rabbit IgG H&L horseradish peroxidase (HRP)-conjugated secondary antibody at 37 °C for 1 h. Finally, tissue sections were stained with 3,3′-diaminobenzidine (DAB) and subsequently hematoxylin at room temperature. Images were captured using a photomicroscope (Olympus, Shinjuku, Tokyo, Japan) and analyzed. The staining intensity was scored as 0 (negative), 1 (weak), or 2 (strong). The staining extent was graded as 0 (0%), 1 (1–25%), 2 (26–50%), 3 (51–75%), or 4 (76–100%). The samples were classified into high and low expression groups based on the sum of staining intensity and extent. Antibodies are listed in Table S2.

### Detection of serum leptin level

Blood samples were drawn from cohort 2 before operation. The samples were separated through centrifugation at 800 × *g* for 30 min. The serum concentrations of leptin were determined by Leptin Human ELISA Kit (# KAC2281, Thermo Fisher Scientific, Waltham, MA, USA) according to the manufacturer’s protocol.

### Vectors construction and lentivirus transfection

For over-expression, the MPS-1 construct was generated by subcloning human MPS-1 cDNA into vector BR-V-214 (Shanghai Biosciences, Shanghai, China). Lentivirus production and transfection were performed as previously described^14^. For gene knockdown, target short hairpin RNA (shRNA) sequence or scramble shRNA (shCtrl) was packaged into vector BR-V-215 (Shanghai Biosciences) using Fermentas T4 DNA Ligase (New England Biolabs). Plasmids containing target shRNA sequences were extracted with EndoFree Plasmid Maxi Kit (Qiagen, Valencia, CA, USA). Cell transfection was conducted with Lipofectamine 2000 (Thermo Fisher Scientific). The cells were screened under Puromycin (Takara Bio, Otsu, Japan) and verified by detecting GFP fluorescence with a fluorescence microscope (Olympus, Tokyo, Japan). The target sequences and corresponding shRNA sequences are listed in Table S3.

### CCK8 assay

Cell proliferation was measured with the Cell Counting Kit-8 (Dojindo, Kumamoto, Japan) according the instruction of manufacturer. Briefly, RKO, HCT116, and Caco2 cells were seeded in 96-well plates with 100 μL medium (3000 cells/well). The measurement was performed at 1, 2, 3, 4, and 5 days after seeding. After CCK-8 reagent was added (10 μL/well), the cells were cultured at 37 °C until visual color conversion occurred. The absorbance was determined at 450 nm with a microplate reader (Tecan, Männedorf, Zürich, Switzerland). Inhibition rate was calculated by the equation: [OD (shCtrl)–OD (shGene)]/OD (shCtrl).

### Colony formation assay

Four days after cell transfection, RKO, HCT116, and Caco2 cells in logarithmic growth phase were digested by trypsin, and seeded in six-well plates (500 cells/well). Cells were cultured in the medium at 37 °C for 14 days to form colonies. Then cells were washed by PBS, fixed by 4% paraformaldehyde for 1 h, stained with Giemsa (500 μL) for 20 min, and photographed with a digital camera. The number of colonies (>50 cells/colony) was counted under fluorescence microscopy (MicroPublisher 3.3 RTV, Olympus, Tokyo, Japan).

### Cell apoptosis flow cytometry assay

The quantitative assessment of apoptosis was performed with flow cytometry. Briefly, RKO, HCT116, and Caco2 cells were plated on six-well plates at a density of 2 × 10^5^ cells/well to assess apoptosis. After incubation for 24 h, cells were harvested and pelleted by centrifugation for Annexin V staining with a eBioscience Annexin V Apoptosis Detection Kit APC (Invitrogen, Carlsbad, CA, USA). Cell pellets were washed twice with phosphate buffer and re-suspended in 250 μL-binding buffer at a density of 1 × 10^6^ cells/mL. The cells were stained with 5 μL Annexin V and incubated on ice for 15 min. The cell suspension (100 μL) was analyzed by flow cytometry to detect apoptosis.

### Caspase 3/7 activity

Apoptotic cell death was quantified by using the Caspase-Glo^®^ 3/7 assay kit (Promega, Madison, WI, USA) according to the manufacturer’s protocol. Briefly, RKO, HCT116, and Caco2 cells at 100,000 cells/well were seeded into 96-well plates. Before detection, 100 μL of Caspase-Glo reagent was added to each well and incubated for 2 h. The luminescence was measured with a microplate reader (Tecan Infinite, Groedig, Austria).

### Mice xenograft model

BALB/c male nude mice (6 weeks of age) were purchased from Shanghai Jiesijie Experimental Animals Co., Ltd (Shanghai, China). Mice were randomly divided into shMPS-1 group and shCtrl group (6 mice/group). RKO cells (5 × 10^6^ cells/mouse) transfected with shMPS-1 or shCtrl were subcutaneously injected into mice. Tumor growth was measured in volume (calculated by the equation *π*/6 × *L* × *W*^2^, where *L* represents longest dimension and *W* means dimension perpendicular to length) on days 6, 12, 18, 24 post inoculation. Mice were sacrificed 25 days after injection and tumor weights were measured. All procedures were approved by the Institutional Animal Care and Use Committee of Shanghai Jiao Tong University.

### Statistical analysis

Results were presented as mean ± SD. Chi-square test was used for categorical variables. Continuous variables were compared with Student’s *t*-test (two groups) or One-way ANOVA followed by Bonferroni post-hoc test (three or more groups). Linear regression analysis was used to evaluate the relationship between MPS-1 and serum leptin level. Kaplan–Meier survival curves with log-rank test was used to analyze overall survival (OS). Each experiment was repeated three times. *P* < 0.05 was defined as statistically significant (two-sided). Statistical analysis was performed using SPSS 14.0 for Windows (SPSS Inc., Chicago, IL, USA).

## Results

### MPS-1 expression was identified as a promoter in CRC, especially in obese CRC patients

**Fig. 1:**
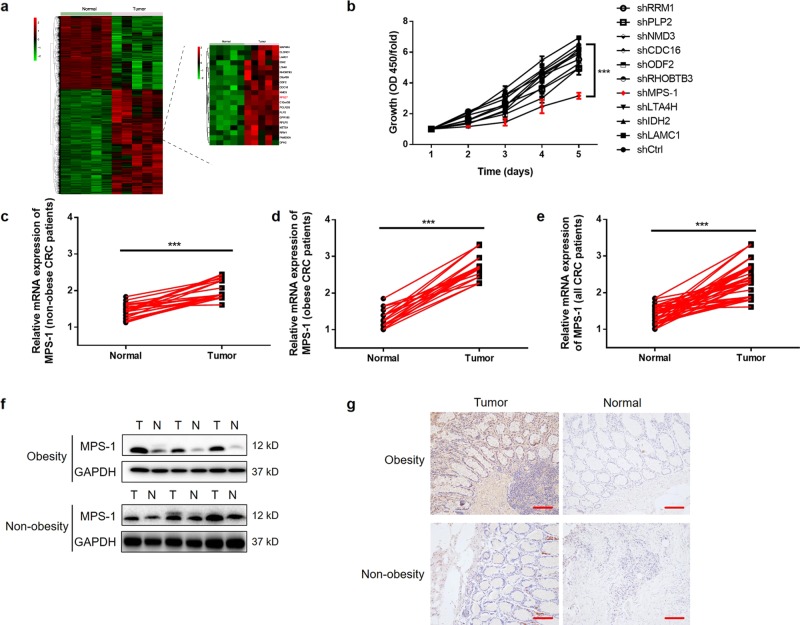
MPS-1 was up-regulated in CRC patients, especially in the context of obesity. **a** Microarray analysis was performed in tumor and normal tissues from cohort 1 to identify differentially expressed genes. **b** The inhibition rate of cell proliferation by silencing the gene candidates was evaluated with CCK8 assay in RKO cells. **c** The mRNA levels of MPS-1 were detected by qPCR in 20 matched pairs of CRC tissues and normal tissues collected from non-obese CRC patients from cohort 2. **d** The mRNA levels of MPS-1 were detected by qPCR in 20 matched pairs of CRC tissues and normal tissues collected from obese CRC patients from cohort 2. **e** The overall demonstration of **c** and **d**. **f** Representative images of MPS-1 protein levels in CRC and normal tissues by WB. **g** Representative images of MPS-1 protein levels in CRC and normal tissues by IHC staining (scale bar = 100 μm). **P < 0.01

To explore novel regulators in CRC, gene expression profiles in five pairs of tumor and normal tissues from cohort 1 were detected by microarray analysis. Transcriptome profiling identified 1140 up-regulated and 1091 down-regulated genes in CRC tissues (Fig. 1a, Table S4). Subsequently, the inhibition impacts of 10 up-regulated candidates on the proliferation of RKO cells were detected by gene knockdown experiments. The results indicated that MPS-1 displayed the largest inhibition rate on cell proliferation (*P* < 0.05, Fig. 1b, Table S4).

To validate the findings in microarray screening, MPS-1 expression was determined in 40 pairs of tumor and normal tissues from cohort 2. Despite the up-regulation in all patients (*P* < 0.001, Fig. 1c–e), MPS-1 mRNA levels showed a higher fold up-regulation in obese CRC patients than in their non-obese counterparts (1.40-fold in non-obese group, 2.12-fold in obese group, Fig. 1c, d). The results of WB and IHC demonstrated similar results, as shown by the representative images in Fig. 1f, g. Notably, the analysis of MPS-1 expression and basic characteristics of cohort 3 found an association between MPS-1 expression and advanced tumor stage (*P* < 0.001, Table 1). These findings suggested MPS-1 as a promoter in the development and progression of CRC.

**Table 1:** Relationship between MPS-1 expression and characteristics of patients of cohort 3

Features	No. of patients	MPS-1 expression		*P-*value
		low	high	
All patients	155	87	68	
Age (years)				0.564
<65	78	42	36	
≥65	77	45	32	
Gender				0.401
Male	83	44	39	
Female	72	43	29	
BMI				0.647
<30	83	48	35	
≥30	72	39	33	
Clinical stage				<0.001***
I	22	19	3	
II	61	28	33	
III	67	40	27	
IV	5	0	5	

***P < 0.001

### MPS-1 expression was correlated with poor prognosis and circulating leptin level in obese CRC patients

**Fig. 2:**
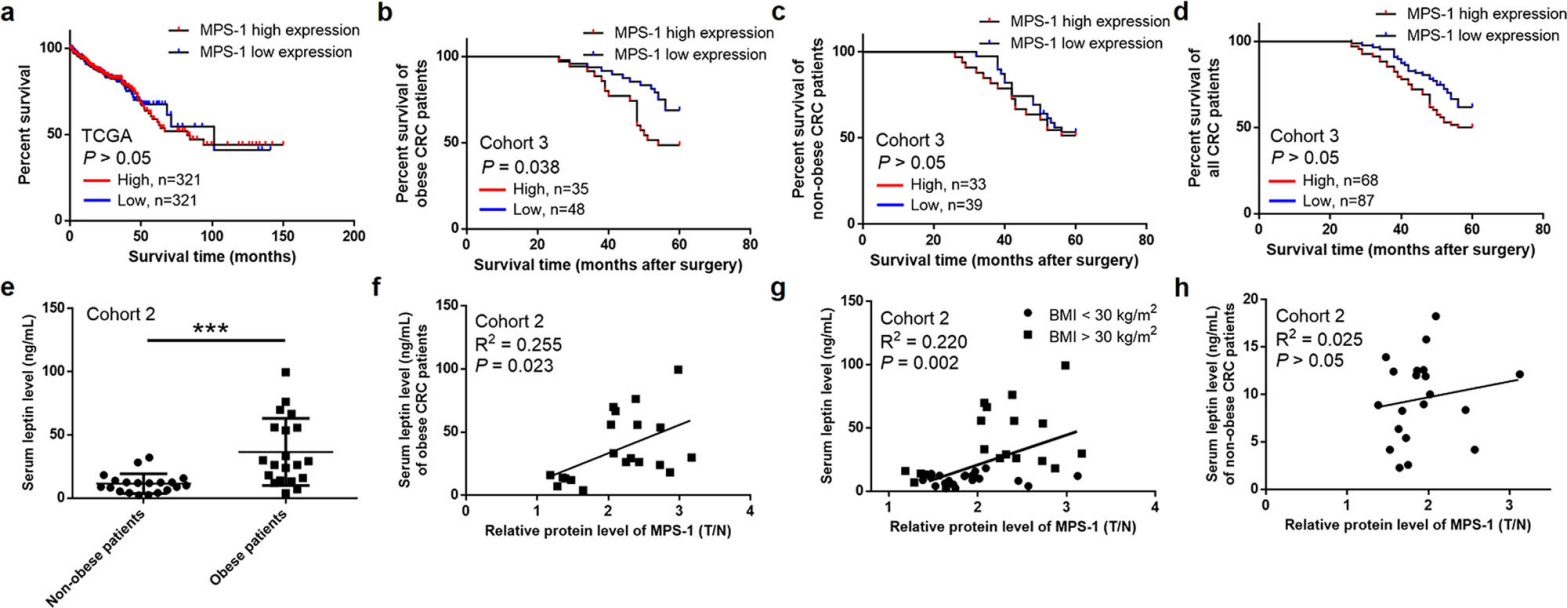
MPS-1 expression was correlated with poor prognosis and circulating leptin level of CRC patients. **a** Kaplan–Meier analysis of the correlation between MPS-1 expression and OS in CRC patients through data mining of TCGA. **b** Kaplan–Meier analysis of the correlation between MPS-1 expression and OS in obese CRC patients from cohort 3. **c** Kaplan–Meier analysis of the correlation between MPS-1 expression and OS in non-obese CRC patients from cohort 3. **d** The overall demonstration of **b** and **c**. **e** Serum levels of leptin were determined by ELISA in the CRC patients of cohort 2. **f** and **g** The correlation between MPS-1 expression and serum leptin levels was analyzed in obese CRC patients **f**, all CRC patients **g** and non-obese CRC patients **h** from cohort 2. ***P < 0.001

In order to determine the relationship between MPS-1 and prognosis of CRC, its impact on OS was preliminarily investigated through data mining of The Cancer Genome Atlas (TCGA). No correlation was found between MPS-1 expression and OS (*P* > 0.05, Fig. 2a). Furthermore, we performed subgroup analysis in cohort 3 according to BMI. Kaplan–Meier analysis indicated that MPS-1 over-expression was correlated with poor OS in obese CRC patients (*P* = 0.038, Fig. 2b) instead of non-obese cases (*P* > 0.05, Fig. 2c), which might contribute to the statistical insignificance in survival analysis of all patients (*P* > 0.05, Fig. 2d).

Moreover, the relationship between MPS-1 and serum leptin was analyzed to investigate its role in obese CRC patients. With a significant overall correlation (*P* < 0.001, Fig. 2e), a positive correlation was found between MPS-1 expression and leptin level in obese CRC patients (*R*^2^ = 0.255, *P* = 0.023, Fig. 2f) and in all CRC patients from cohort 2 (*R*^2^ = 0.220, *P* = 0.002, Fig. 2g) but not in non-obese cases (*R*^2^ = 0.025, *P* > 0.05, Fig. 2h). These observations suggested that MPS-1 might be implicated in obesity-associated CRC through interacting with leptin.

### MPS-1-induced tumor growth and suppressed apoptosis in CRC cells

**Fig. 3:**
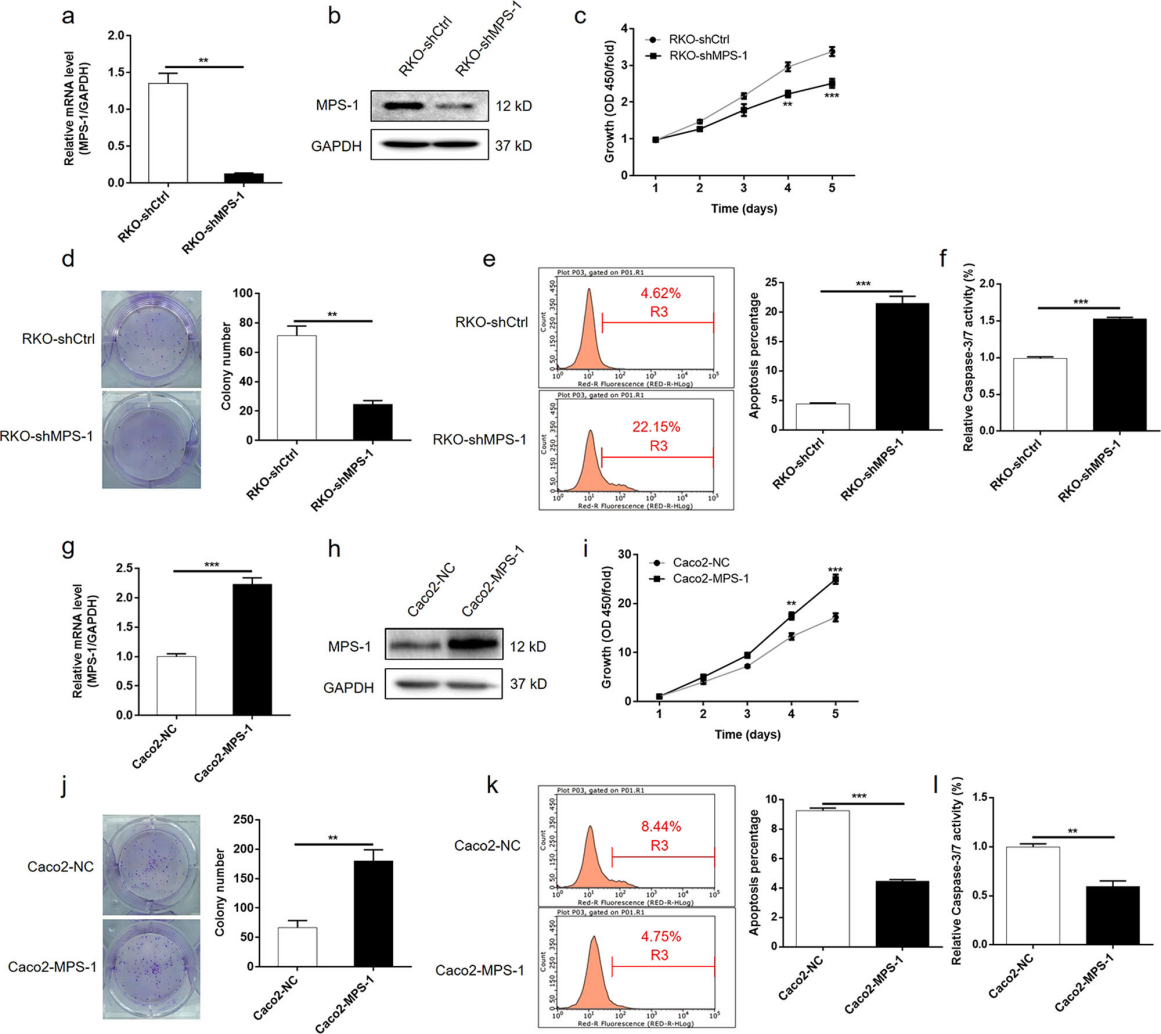
MPS-1-regulated cell proliferation and apoptosis of CRC cells. **a**, **b** The efficiency of MPS-1 gene knockdown in RKO cells was evaluated by qPCR **a** and WB **b**. **c**–**f** MPS-1 gene knockdown suppressed cell proliferation **c** and colony formation **d**, and induced apoptosis **e** and activity of Caspase 3/7 **f** in RKO cells. **g**, **h** The efficiency of MPS-1 gene over-expression in Caco2 cells was evaluated by qPCR **g** and WB **h**. **i**–**l** Over-expression of MPS-1 in RKO cells promoted cell proliferation **i** and colony formation **j**, and suppressed apoptosis **k** and activity of Caspase 3/7 **l**. Error bars indicate SD of three biological replicates. *P < 0.05, **P < 0.01, ***P < 0.001

Next, the impact of alteration in MPS-1 levels on CRC cells was investigated in vitro. As shown in Fig. S1a and S1b, the expression of MPS-1 in CRC cells was up-regulated compared with normal colon cell FHC (*P* < 0.01), suggesting its role as a promoter in CRC. Subsequently, MPS-1 gene was knockdown in RKO cells and over-expressed in Caco2 cells, respectively. The alteration in the expression of MPS-1 was verified at mRNA and protein levels (*P* < 0.01, Fig. 3a, b, g, h, S2a, S2b). As shown in Figs. 3c, d and S2c, S2d, the knockdown of MPS-1 suppressed cell proliferation (25.6% inhibition rate for RKO cells, *P* < 0.01; 24.4% inhibition rate for HCT116 cells, *P* < 0.01) and colony formation (65.4% inhibition rate for RKO cells, *P* < 0.01; 69.1% inhibition rate for HCT116 cells, *P* < 0.001) compared with the negative control. Moreover, the down-regulation of MPS-1 promoted apoptosis in CRC cells (4.88-fold in RKO cells, *P* < 0.001; 4.72-fold in HCT116 cells, *P* < 0.001) (Figs. 3e and S2e). Besides, enhanced activity of Caspase 3/7 was detected (1.54-fold in RKO cells, *P* < 0.001; 2.04-fold in HCT116 cells, *P* < 0.001), which may be an explanation for the enhanced apoptosis (Figs. 3f and S2f). Consistent with this, the over-expression of MPS-1 showed exactly conversed effect on CRC cells (*P* < 0.01, Fig. 3i–l). Collectively, the results demonstrated that MPS-1 plays a promotion role in the development of CRC.

### Leptin alleviated the inhibition effect of MPS-1 knockdown on CRC cells

**Fig. 4:**
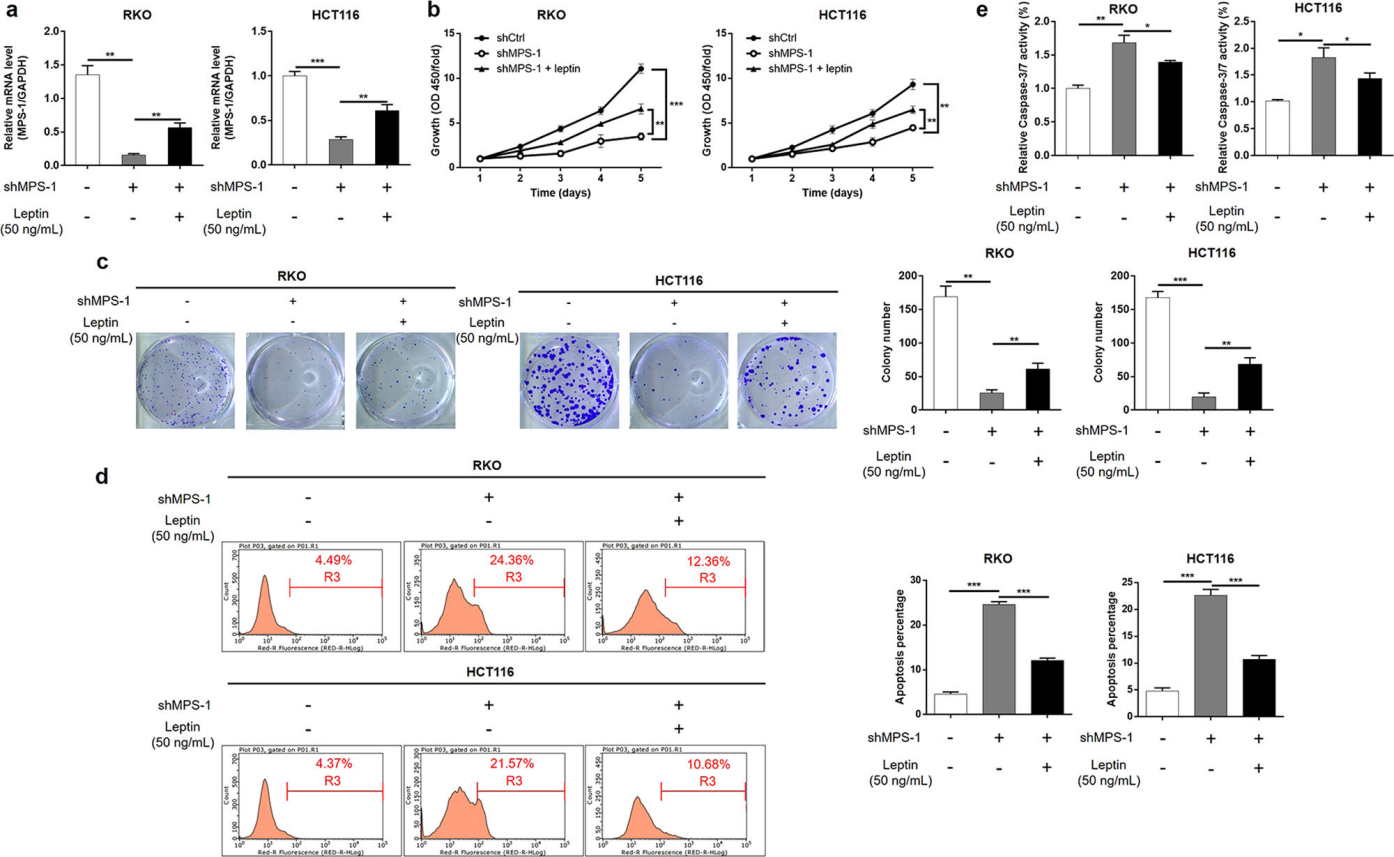
The suppression effect of MPS-1 knockdown on CRC development was alleviated by leptin. **a** The impact of leptin (50 ng/mL) on MPS-1 expression in RKO and HCT116 cells with MPS-1 knockdown was detected by qPCR. **b** The effect of leptin on cell proliferation of RKO and HCT116 cells with MPS-1 knockdown was detected by CCK8 assay. **c** The effect of leptin on colony formation of RKO and HCT116 cells with MPS-1 knockdown was detected by colony formation assay. **d** The effect of leptin on apoptosis of RKO and HCT116 cells with MPS-1 knockdown was detected by flow cytometry. **e** The effect of leptin on Caspase-3/7 activity was detected in RKO and HCT116 with MPS-1 knockdown. Error bars indicate SD of three biological replicates. *P < 0.05, **P < 0.01, ***P < 0.001

Given that alterations in MPS-1 expression predominantly occurred in obese CRC patients, and MPS-1 expression was correlated with serum leptin in obese CRC patients, we focused our attention on the relationship between MPS-1 and leptin. The impact of leptin on CRC cells with MPS-1 knockdown was evaluated with pharmacologic enhancement of leptin. The results of qPCR proved that treatment of leptin partially reverted the effect of MPS-1 gene knockdown (34.07% recovery for RKO, 45.26% recovery for HCT116, *P* < 0.01, Fig. 4a). Subsequently, it was observed that leptin exposure partially reversed the effect of MPS-1 knockdown on cell proliferation (40.68% recovery for RKO, 41.46% recovery for HCT116) and colony formation (25.01% recovery for RKO, 32.87% recovery for HCT116) in both RKO and HCT116 cells (*P* < 0.01, Fig. 4b, c). Additionally, leptin exposure significantly alleviated the promotion effect of MPS-1 knockdown on cell apoptosis (62.34% recovery for RKO, 66.76% recovery for HCT116) and Caspase-3/7 activity (42.63% recovery for RKO, 48.39% recovery for HCT116) in RKO and HCT116 cells (*P* < 0.05, Fig. 4d, e). Taken together, the data demonstrated that MPS-1 modulated the development of CRC under the regulation of leptin.

### MPS-1 promoted CRC through activation of JNK/c-Jun-signaling pathway

**Fig. 5:**
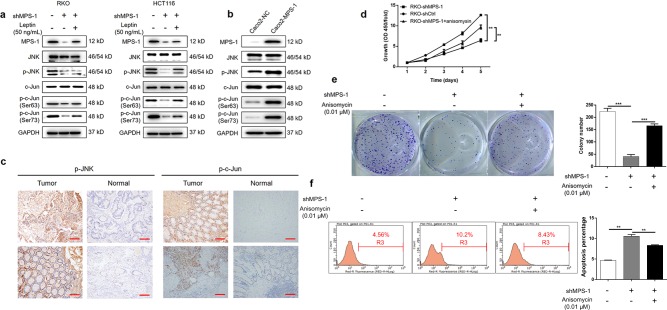
MPS-1 mediated leptin-associated CRC progression via activation of JNK/c-Jun pathway. **a** The protein levels of MPS-1, JNK, p-JNK, c-Jun, and p-c-Jun in RKO and HCT116 cells treated with shCtrl or shMPS-1 or shMPS-1 + leptin (50 ng/mL) were detected by WB. **b** The protein levels of MPS-1, JNK, p-JNK, c-Jun, and p-c-Jun in Caco2 cells with or without over-expression of MPS-1 were detected by WB. **c** The protein levels of p-JNK and p-c-Jun were detected in CRC tissues by IHC staining (scale bar = 100 μm). **d** Cell proliferation was detected by CCK8 in RKO cells treated with shCtrl or shMPS-1 or shMPS-1 + anisomycin (JNK activator, 0.01 μM). **e** Colony formation was evaluated in RKO cells treated with shCtrl or shMPS-1 or shMPS-1 + anisomycin (0.01 μM). **f** Apoptosis was evaluated in RKO cells treated with shCtrl or shMPS-1 or shMPS-1 + anisomycin (0.01 μM). Error bars indicate SD of three biological replicates. *P < 0.05, **P < 0.01, ***P < 0.001

In order to investigate the mechanisms of the promotion effect of MPS-1 on CRC, protein levels of JNK/c-Jun pathway including JNK, p-JNK, c-Jun, and p-c-Jun (Ser-63/73) were measured by WB. As shown in Fig. 5a, the activities of JNK and c-Jun were suppressed as illustrated by the down-regulation in p-JNK and p-c-Jun expressions. Conversely, over-expression of MPS-1-enhanced activities of both JNK and c-Jun compared with the negative control in Caco2 cells (Fig. 5b). In consistent, the expressions of p-JNK and p-c-Jun detected by IHC staining in clinical specimens showed generally higher levels in tumors than in the normal tissues (Fig. 5c). In addition, the inhibition effect on JNK/c-Jun signaling of MPS-1 knockdown was partially recovered in response to leptin exposure (Fig. 5a). Further detection showed that the effects of MPS-1 knockdown on proliferation (52.73% recovery), colony formation (68.31% recovery), and apoptosis (37.63% recovery) of RKO cells were significantly compromised by treatment of the JNK activator, anisomycin (*P* < 0.01, Fig. 5d–f). The results indicated that MPS-1 mediated leptin-induced development of CRC via activation of JNK/c-Jun-signaling pathway.

### MPS-1 silencing suppressed tumor formation of CRC in xenografts

**Fig. 6:**
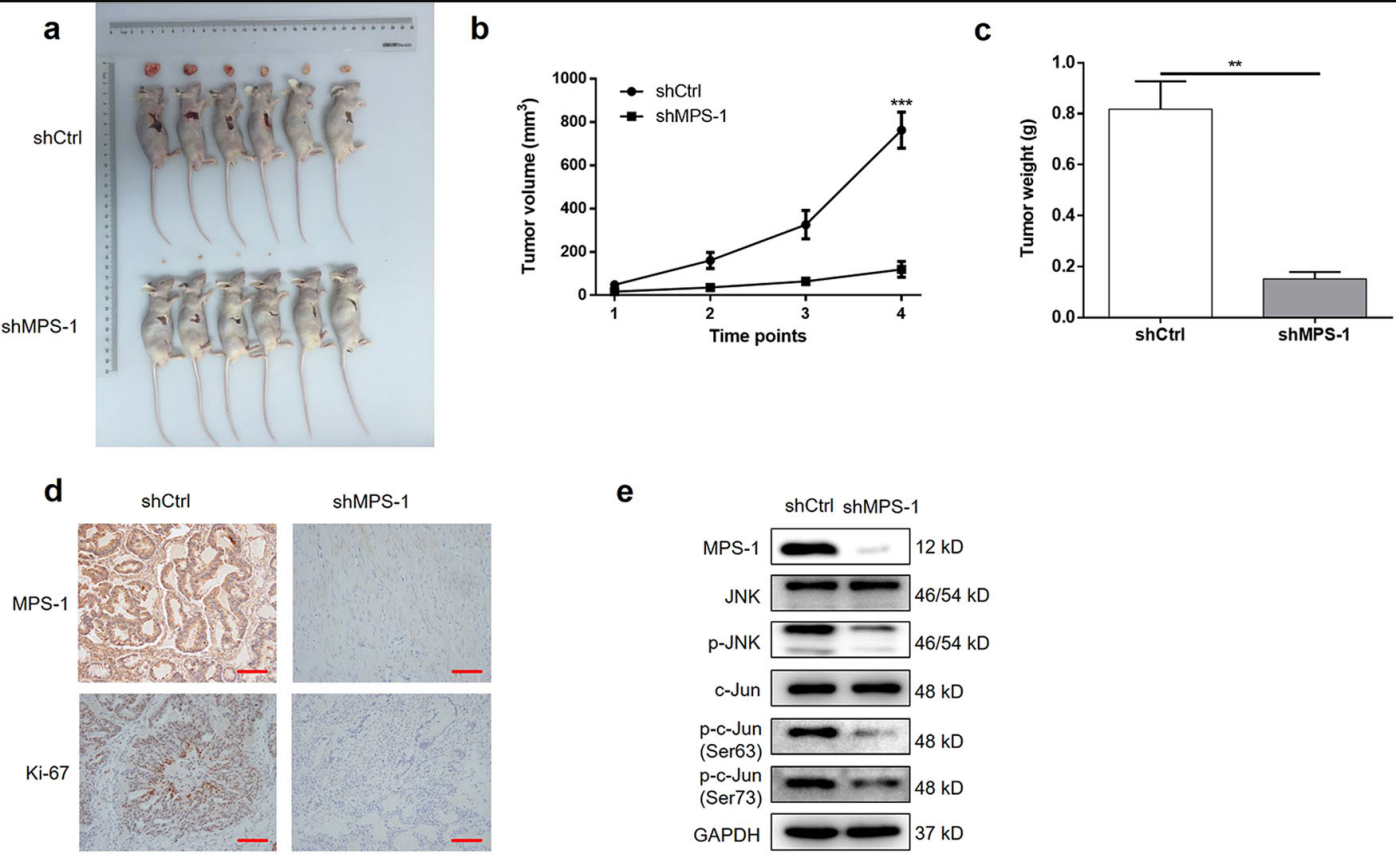
MPS-1 knockdown suppressed tumor growth in vivo. **a** The tumor images taken from mice in shCtrl and shMPS-1 groups. **b** Tumor volume was measured in shCtrl and shMPS-1 groups at the indicated time points (6, 12, 18, 24 days post-injection). **c** Tumor weights were measured in shCtrl and shMPS-1 groups after sacrificing mice. **d** The levels of MPS-1 and Ki-67 were analyzed by IHC staining (scale bar = 100 μm). **f** The protein expressions of MPS-1, JNK, p-JNK, c-Jun, and p-c-Jun (Ser63 and Ser73) were detected by WB in the tumors of shCtrl and shMPS-1 groups. Error bars indicate SD of three biological replicates. *P < 0.05, **P < 0.01, ***P < 0.001

Lastly, to confirm that the in vitro findings are relevant in vivo, RKO cells transfected with shMPS-1 or shCtrl were injected into mice, respectively, for constructing mice xenograft model. As shown in Fig. 6a–c, mice implanted with RKO cells with MPS-1 knockdown exhibited much slower growth and smaller size of tumors (84.29% inhibition of volume, 81.42% inhibition of weight, *P* < 0.01). In consistent, the tumors formed in shMPS-1 group displayed lower Ki-67 expression than those in shCtrl group (Fig. 6d). In addition, the suppression of activities of JNK and c-Jun was observed in shMPS-1 group, since expressions of p-JNK and p-c-Jun were down-regulated (Fig. 6e). This indicated that MPS-1 knockdown suppressed tumor formation of CRC via suppressing JNK/c-Jun-signaling pathway. Thus, in vivo experiments confirmed that MPS-1 played a crucial role in tumor progression of CRC via activation of JNK/c-Jun-signaling pathway.

## Discussion

CRC ranks the third most frequently diagnosed cancer worldwide, contributing to a crucial cause of cancer-related death and substantial socioeconomic burden^15^. Although a growing number of therapeutic strategies have been developed over the past decades, the prognosis of CRC is still poor and frustrating^16^. Hence, in-depth investigation into underlying mechanisms is needed to combat the relapse of CRC and improve the prognosis.

In this study, we identified MPS-1 as a candidate promotor in CRC through microarray and gene knockdown experiments. Meanwhile, correlation analysis showed an association between MPS-1 expression and advanced tumor stage, suggesting its association with CRC progression and the potential as a prognostic marker. Previous studies showed variable roles of MPS-1 in the different cancer types. For example, while MPS-1 was a tumor suppresser in head and neck squamous cell carcinoma^6^, MPS-1 over-expression was observed in breast cancer^7,8^ and gastric cancer^9–11^. Until now, little was known on the role of MPS-1 in CRC. The only literature was reported by Ganger et al. illustrating a relationship between MPS-1 over-expression in colonic mucosa crypts and the aggressive behavior of CRC in a patient^12^. However, evidence from cohort study and functional experiment is lacking and warranted. Interestingly, another member of RPS27 family, RPS27a, is highly expressed in CRC and supposed as an early response gene, which might be an indirect support for the implication of MPS-1 in CRC^17^. The current study exhibited that MPS-1 functions to induce the proliferation and suppress apoptosis in CRC cells in vitro. In vivo experiments demonstrated significant suppression in tumor growth by MPS-1 knockdown. In consistent, Ki-67 expression was decreased in the tumors formed in shMPS-1 group, indicating a lower tumor proliferative fraction in this situation. These results demonstrated that MPS-1 plays a promotion role in CRC development.

Obesity is widespread recognized as a major epigenetic cause for CRC^1^. Since obesity is a global epidemic and is predicted to continue to increase, it is important to dissect the mechanisms behind to prevent the increasing trend in obesity-associated CRC. Among the regulatory pathways, the complex role of adipokines (such as leptin and adiponectin) secreted by excess adipose tissue is a focus for research^1,18^. In the current study, the up-regulation of MPS-1 expression was observed by microarray screening and IHC verification in clinical specimens. However, data mining of TCGA showed no statistical significance in survival analysis. Intriguingly, in the subgroup analysis according to BMI, MPS-1 was associated with poor prognosis in obese CRC patients, but not in their non-obese counterparts. Subgroup analysis also found a higher fold up-regulation of MPS-1 expression in tumor tissues compared with corresponding normal tissues in the context of obesity. Therefore, we hypothesized an association between MPS-1 and obesity-driven CRC development, and focused on this compartment.

MPS-1 is a growth factor-inducible gene, but its upstream regulator is unclear. In this study, we revealed that leptin interferes with the effects of MPS-1 in the progression of CRC. Leptin increases in proportion to the level of obesity^3^, and has profound effects in various cancers^14^. For example, Garofalo et al. reported the up-regulation of leptin and its receptor in breast cancer, and identified leptin as a biomarker for tumor progression^19^. It was also demonstrated that leptin promoted the development of lung cancer via JAK/STAT3 pathway^13,20^. Importantly, epidemiological data has established a link between leptin over-expression and high risk of CRC^21^. Moreover, the promotion function of leptin on growth and migration of CRC has been elucidated^22–25^. In consistent, we found that MPS-1 was correlated with circulating leptin level in CRC patients, especially in obese cases, but not in non-obese cases. In addition, the influence of MPS-1 knockdown on CRC cells was alleviated in response to leptin exposure. Our findings identify MPS-1 as a new player in promoting the development of leptin-mediated CRC.

In this study, we found that MPS-1 silencing inhibited cell growth and induced apoptosis of CRC cells by suppressing JNK/c-Jun pathway, while MPS-1 over-expression displayed the opposite effects by stimulating the signal transduction pathway. Besides, anisomycin, a JNK activator, reversed the inhibition effects of MPS-1 knockdown. These results indicated that MPS-1 is able to stimulate JNK/c-Jun pathway. JNK belongs to the mitogen-activated protein kinase (MAPK) family, and is activated in response to cellular stress^26^. JNK pathway is extensively involved in malignancies including CRC^27,28^. For example, TASIN-1, an inhibitor of mutant adenomatous polyposis coli (APC) gene, suppresses CRC through cholesterol depletion via JNK pathway^29^. JNK signaling is linked with obesity-driven disorders such as insulin resistance and chronic inflammation^30^. Endo et al. found higher JNK activity in the mice fed with high fat diet (HFD) than in normal diet (ND) group. The effect of JNK on proliferation of colon cells could be suppressed under HFD but not ND, suggesting its role in obesity-associated CRC^31^. Recent evidences support the involvement of JNK signaling in leptin-related cancers such as ovarian cancer^32^, prostate cancer^33^ and CRC^23^. However, little is known about the exact mechanism. The current study demonstrated that MPS-1 exerts its action in leptin-associated CRC via JNK/c-Jun pathway. Our findings further advanced the understanding of leptin-mediated CRC.

There are some limitations which should be taken into account in the interpretation of these results. First, cohort 1 for microarray analysis has a small sample size. The subsequent analysis in cohort 2 and cohort 3, however, established a correlation between MPS-1 and leptin-induced CRC progression. Second, the mechanism underlying the interaction between MPS-1 and leptin needs further exploration. Third, it is noteworthy that obesity is associated with various adipokines besides leptin. So far, little is known on the regulatory effects of other adipokines on the function of MPS-1 in CRC. In the future, further studies should be conducted for detailed functional assessment of MPS-1 in leptin-mediated CRC.

**Fig. 7:**
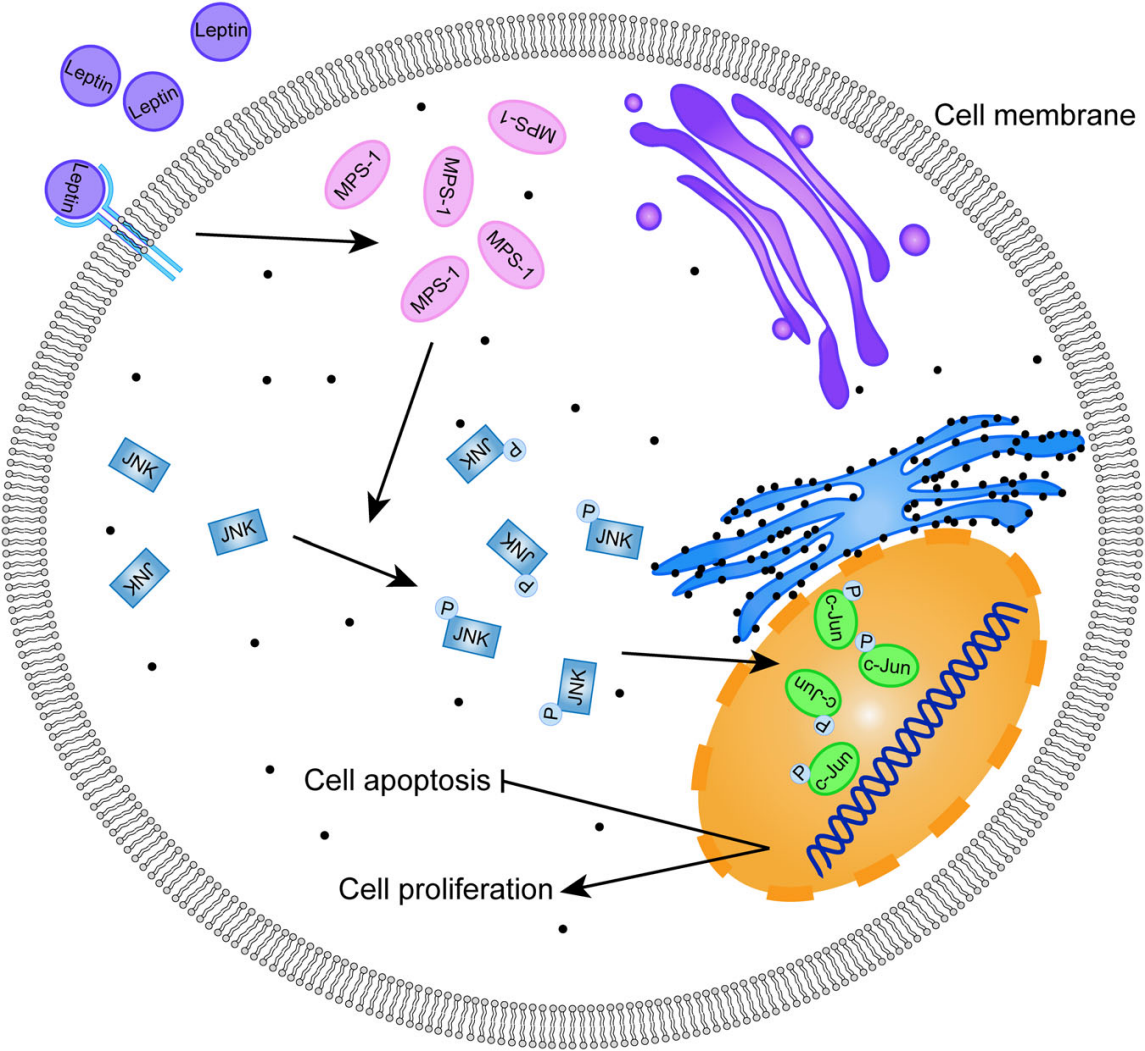
Schematic diagram of MPS-1 mediated leptin-induced CRC via activation of JNK/c-Jun signaling pathway

In conclusion, our findings provide insights of the promoting role of MPS-1 in leptin-induced CRC development via activation of JNK/c-Jun-signaling pathway (Fig. 7). MPS-1 might represent a potent candidate for the treatment and prognostic prediction of CRC patients, especially obese CRC cases.

## Supplementary information

Figure S1

Figure S2

Table S1

Table S2

Table S3

Table S4

Supplemetary figure legends
