# Supplementary figures and images for: Metallopanstimulin-1 (MPS-1) mediates the promotion effect of leptin on colorectal cancer through activation of JNK/c-Jun signaling pathway
Source: Cell Death Dis. 2019 Sep 10;10(9):655. doi: 10.1038/s41419-019-1911-8 (PMC6736844; doi:10.1038/s41419-019-1911-8)

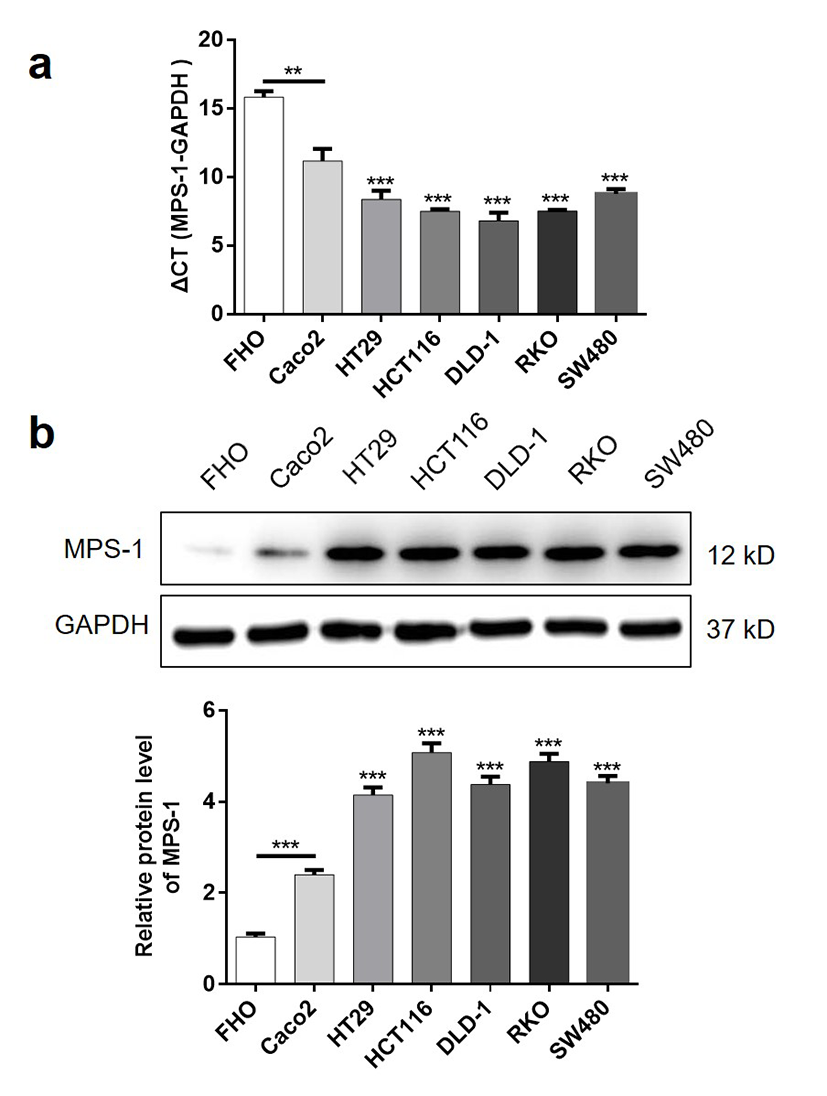

Supplement: Supplementary file 1 — Figure S1 [file 41419_2019_1911_MOESM1_ESM.tif]

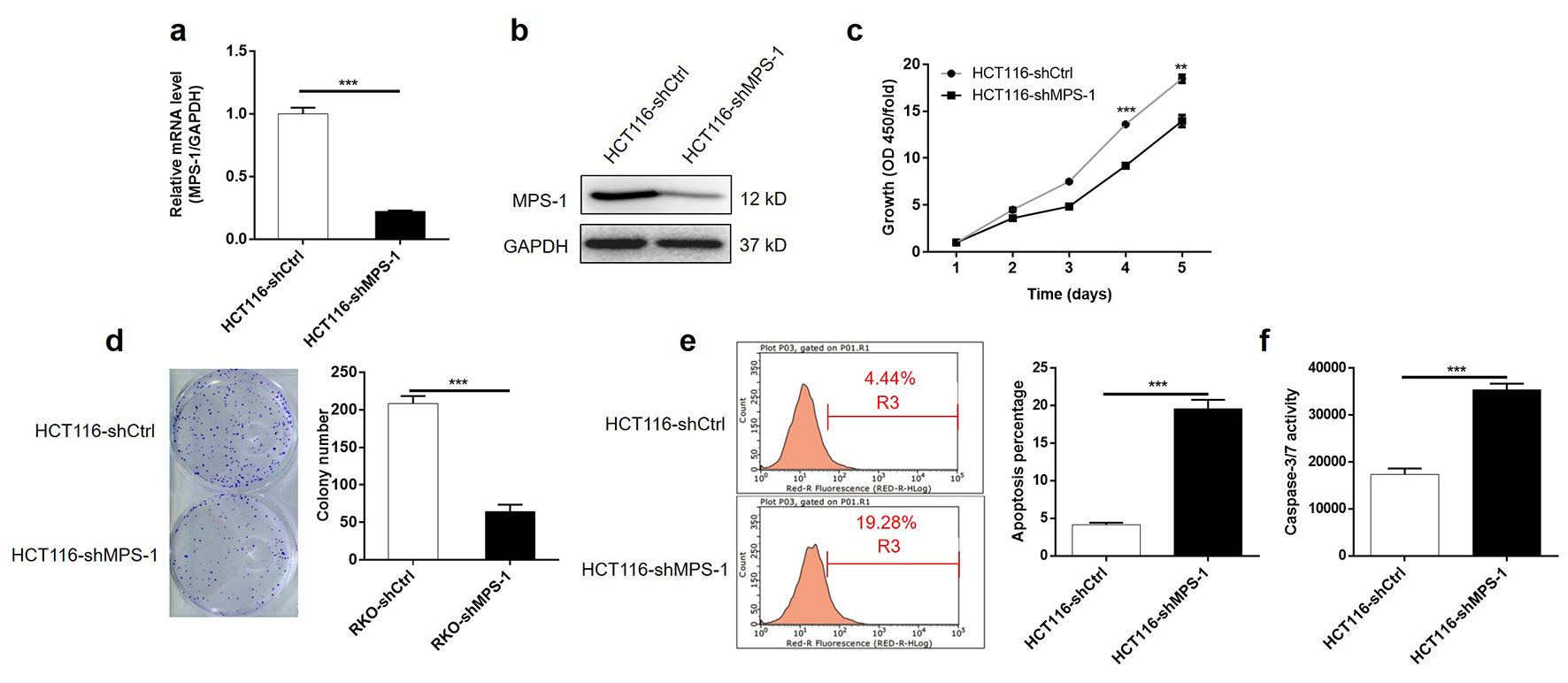

Supplement: Supplementary file 2 — Figure S2 [file 41419_2019_1911_MOESM2_ESM.tif]
