# Supplementary material for: Metallopanstimulin-1 (MPS-1) mediates the promotion effect of leptin on colorectal cancer through activation of JNK/c-Jun signaling pathway
Source: Cell Death Dis. 2019 Sep 10;10(9):655. doi: 10.1038/s41419-019-1911-8 (PMC6736844; doi:10.1038/s41419-019-1911-8)
Supplement: Supplementary file 3 — Table S1 [file 41419_2019_1911_MOESM3_ESM.docx]

Table S1 Primers used for qPCR

|  | sequence (5’-3’) |
| --- | --- |
| MPS-1-F | GATGTGAAATGCCCAGGAT |
| MPS-1-R | GCAGAGGACAGTGGAGCAG |
| GAPDH-F | TGACTTCAACAGCGACACCCA |
| GAPDH-R | CACCCTGTTGCTGTAGCCAAA |
