# Supplementary material for: Metallopanstimulin-1 (MPS-1) mediates the promotion effect of leptin on colorectal cancer through activation of JNK/c-Jun signaling pathway
Source: Cell Death Dis. 2019 Sep 10;10(9):655. doi: 10.1038/s41419-019-1911-8 (PMC6736844; doi:10.1038/s41419-019-1911-8)
Supplement: Supplementary file 4 — Table S2 [file 41419_2019_1911_MOESM4_ESM.docx]

Table S2 Antibodies used in WB and IHC

| Primary antibodies | Description | Reactivity | Dilution | Company and catalog No. |
| --- | --- | --- | --- | --- |
| MPS-1 | Goat polyclonal | Human MPS-1 | 1:1,000 (WB)  1:200 (IHC) | Abcam # ab4385 |
| anti-JNK1+JNK2 | Rabbit polyclonal | Human JNK1+JNK2 | 1:1,000 | Abcam # ab112501 |
| p-JNK | Mouse monoclonal | Human p-JNK | 1:1,000 (WB)  1:300 (IHC) | Santa Cruz Biotechnology # sc-6254 |
| c-Jun | Rabbit monoclonal | Human c-Jun | 1:1,000 | CST # 60A8 |
| p-c-Jun (Ser 73) | Rabbit monoclonal | Human p-c-Jun (Ser 73) | 1:1,000 (WB)  1:250 (IHC) | CST # 3270s |
| p-c-Jun (Ser 63) | Rabbit monoclonal | Human p-c-Jun (Ser 63) | 1:1,000 | Abcam # ab32385 |
| GAPDH | Rabbit polyclonal | Human GAPDH | 1:3,000 | Bioworld # AP0063 |
| Ki-67 | Rabbit monoclonal | Human Ki-67 | 1:200 (IHC) | Abcam # ab16667 |
| Leptin Receptor | Rabbit polyclonal | Human leptin receptor | 1:2,000 | Abcam # ab5593 |
| Secondary antibody |  |  |  |  |
| HRP Goat Anti-Rabbit IgG |  |  | 1:3,000 (WB)  1:400 (IHC) | Abcam # ab6721 |
| HRP Goat Anti-Mouse IgG |  |  | 1:1,000 (WB)  1:50 (IHC) | Beyotime # A0216 |
| HRP Donkey Anti-Goat IgG |  |  | 1:1,000 (WB)  1:50 (IHC) | Beyotime # A0181 |
