# Supplementary material for: Metallopanstimulin-1 (MPS-1) mediates the promotion effect of leptin on colorectal cancer through activation of JNK/c-Jun signaling pathway
Source: Cell Death Dis. 2019 Sep 10;10(9):655. doi: 10.1038/s41419-019-1911-8 (PMC6736844; doi:10.1038/s41419-019-1911-8)
Supplement: Supplementary file 5 — Table S3 [file 41419_2019_1911_MOESM5_ESM.docx]

Table S3 The target sequences and shRNA sequences

| Gene | No. | Target sequence | shRNA sequences (5'-3') |
| --- | --- | --- | --- |
| RRM1 | Pbr-12073 | GTGCACAGAAATAGTGGAGTA | ccggGTGCACAGAAATAGTGGAGTActcgagTACTCCACTATTTCTGTGCACtttttg |
|  |  |  | aattcaaaaaGTGCACAGAAATAGTGGAGTActcgagTACTCCACTATTTCTGTGCAC |
| RRM1 | Pbr-12074 | AAGGACAAGACCAGCGGCTAA | ccggAAGGACAAGACCAGCGGCTAActcgagTTAGCCGCTGGTCTTGTCCTTtttttg |
|  |  |  | aattcaaaaaAAGGACAAGACCAGCGGCTAActcgagTTAGCCGCTGGTCTTGTCCTT |
| RRM1 | Pbr-12075 | CATGAAACGAGTGGAGACTAA | ccggCATGAAACGAGTGGAGACTAActcgagTTAGTCTCCACTCGTTTCATGtttttg |
|  |  |  | aattcaaaaaCATGAAACGAGTGGAGACTAActcgagTTAGTCTCCACTCGTTTCATG |
| CDC16 | Pbr-28779 | CATGGAAGAGCCCATCAATAA | ccggCATGGAAGAGCCCATCAATAActcgagTTATTGATGGGCTCTTCCATGtttttg |
|  |  |  | aattcaaaaaCATGGAAGAGCCCATCAATAActcgagTTATTGATGGGCTCTTCCATG |
| CDC16 | Pbr-28780 | TTCTGTAGTAATGGAGAAAGA | ccggTTCTGTAGTAATGGAGAAAGActcgagTCTTTCTCCATTACTACAGAAtttttg |
|  |  |  | aattcaaaaaTTCTGTAGTAATGGAGAAAGActcgagTCTTTCTCCATTACTACAGAA |
| CDC16 | Pbr-28781 | AGCTTTGAAGCTTGATGTCTA | ccggAGCTTTGAAGCTTGATGTCTActcgagTAGACATCAAGCTTCAAAGCTtttttg |
|  |  |  | aattcaaaaaAGCTTTGAAGCTTGATGTCTActcgagTAGACATCAAGCTTCAAAGCT |
| LTA4H | Pbr-23775 | CAGGACACTCCTTCTGTGAAA | ccggCAGGACACTCCTTCTGTGAAActcgagTTTCACAGAAGGAGTGTCCTGtttttg |
|  |  |  | aattcaaaaaCAGGACACTCCTTCTGTGAAActcgagTTTCACAGAAGGAGTGTCCTG |
| LTA4H | Pbr-23776 | TTGGAGAAAGACAAAGTTACA | ccggTTGGAGAAAGACAAAGTTACActcgagTGTAACTTTGTCTTTCTCCAAtttttg |
|  |  |  | aattcaaaaaTTGGAGAAAGACAAAGTTACActcgagTGTAACTTTGTCTTTCTCCAA |
| LTA4H | Pbr-23777 | CTGAGAAAGAGCAGGTGGAAA | ccggCTGAGAAAGAGCAGGTGGAAActcgagTTTCCACCTGCTCTTTCTCAGtttttg |
|  |  |  | aattcaaaaaCTGAGAAAGAGCAGGTGGAAActcgagTTTCCACCTGCTCTTTCTCAG |
| IDH2 | Pbr-11370 | CAGTACAAGGCCACAGACTTT | ccggCAGTACAAGGCCACAGACTTTctcgagAAAGTCTGTGGCCTTGTACTGtttttg |
|  |  |  | aattcaaaaaCAGTACAAGGCCACAGACTTTctcgagAAAGTCTGTGGCCTTGTACTG |
| IDH2 | Pbr-11371 | CTGCAAGAACTATGACGGAGA | ccggCTGCAAGAACTATGACGGAGActcgagTCTCCGTCATAGTTCTTGCAGtttttg |
|  |  |  | aattcaaaaaCTGCAAGAACTATGACGGAGActcgagTCTCCGTCATAGTTCTTGCAG |
| IDH2 | Pbr-11372 | TGGGAAGACGATTGAGGCTGA | ccggTGGGAAGACGATTGAGGCTGActcgagTCAGCCTCAATCGTCTTCCCAtttttg |
|  |  |  | aattcaaaaaTGGGAAGACGATTGAGGCTGActcgagTCAGCCTCAATCGTCTTCCCA |
| LAMC1 | Pbr-14188 | TCCTGCAAAGTTCTTGGGCAA | ccggTCCTGCAAAGTTCTTGGGCAActcgagTTGCCCAAGAACTTTGCAGGAtttttg |
|  |  |  | aattcaaaaaTCCTGCAAAGTTCTTGGGCAActcgagTTGCCCAAGAACTTTGCAGGA |
| LAMC1 | Pbr-14189 | ATTTGATAAGCTGGTGTGTAA | ccggATTTGATAAGCTGGTGTGTAActcgagTTACACACCAGCTTATCAAATtttttg |
|  |  |  | aattcaaaaaATTTGATAAGCTGGTGTGTAActcgagTTACACACCAGCTTATCAAAT |
| LAMC1 | Pbr-14190 | CTGTACAAACGCTGTTGGCTA | ccggCTGTACAAACGCTGTTGGCTActcgagTAGCCAACAGCGTTTGTACAGtttttg |
|  |  |  | aattcaaaaaCTGTACAAACGCTGTTGGCTActcgagTAGCCAACAGCGTTTGTACAG |
| MPS-1 | Pbr-10605 | AAACCATCTCAATAAACACAT | CCGGAAACCATCTCAATAAACACATTTCAAGAGAATGTGTTTATTGAGATGGTTTTTTTTG |
|  |  |  | AATTCAAAAAAAACCATCTCAATAAACACATTCTCTTGAAATGTGTTTATTGAGATGGTTT |
| MPS-1 | Pbr-00209 | ACGGTCTTTAGCCATGCACAA | ccggACGGTCTTTAGCCATGCACAATTCAAGAGATTGTGCATGGCTAAAGACCGTtttttg |
|  |  |  | aattcaaaaaACGGTCTTTAGCCATGCACAATTCAAGAGATTGTGCATGGCTAAAGACCGT |
| MPS-1 | Pbr-00210 | GCCCAGGATGCTATAAAATCA | ccggGCCCAGGATGCTATAAAATCATTCAAGAGATGATTTTATAGCATCCTGGGCtttttg |
|  |  |  | aattcaaaaaGCCCAGGATGCTATAAAATCATTCAAGAGATGATTTTATAGCATCCTGGGC |
| PLP2 | Pbr-31515 | TACCTGATCACCTCCATTGTT | ccggTACCTGATCACCTCCATTGTTctcgagAACAATGGAGGTGATCAGGTAtttttg |
|  |  |  | aattcaaaaaTACCTGATCACCTCCATTGTTctcgagAACAATGGAGGTGATCAGGTA |
| PLP2 | Pbr-31516 | CCGTTCGGCAGCCAAGACATA | ccggCCGTTCGGCAGCCAAGACATActcgagTATGTCTTGGCTGCCGAACGGtttttg |
|  |  |  | aattcaaaaaCCGTTCGGCAGCCAAGACATActcgagTATGTCTTGGCTGCCGAACGG |
| PLP2 | Pbr-31517 | CCTGTTTGCTGAGATTATATT | ccggCCTGTTTGCTGAGATTATATTctcgagAATATAATCTCAGCAAACAGGtttttg |
|  |  |  | aattcaaaaaCCTGTTTGCTGAGATTATATTctcgagAATATAATCTCAGCAAACAGG |
| NMD3 | Pbr-31518 | TGCAGCATAGTCCAAGATATA | ccggTGCAGCATAGTCCAAGATATActcgagTATATCTTGGACTATGCTGCAtttttg |
|  |  |  | aattcaaaaaTGCAGCATAGTCCAAGATATActcgagTATATCTTGGACTATGCTGCA |
| NMD3 | Pbr-31519 | TAGAGTAGAAGCTAAGGATTT | ccggTAGAGTAGAAGCTAAGGATTTctcgagAAATCCTTAGCTTCTACTCTAtttttg |
|  |  |  | aattcaaaaaTAGAGTAGAAGCTAAGGATTTctcgagAAATCCTTAGCTTCTACTCTA |
| NMD3 | Pbr-31520 | ATCAGCCAAGGTATTCCGAAA | ccggATCAGCCAAGGTATTCCGAAActcgagTTTCGGAATACCTTGGCTGATtttttg |
|  |  |  | aattcaaaaaATCAGCCAAGGTATTCCGAAActcgagTTTCGGAATACCTTGGCTGAT |
| ODF2 | Pbr-31521 | CAGCTAGAACGCTGTGACAAA | ccggCAGCTAGAACGCTGTGACAAActcgagTTTGTCACAGCGTTCTAGCTGtttttg |
|  |  |  | aattcaaaaaCAGCTAGAACGCTGTGACAAActcgagTTTGTCACAGCGTTCTAGCTG |
| ODF2 | Pbr-31522 | TAAGGCAGAAGTGGAGGCCAT | ccggTAAGGCAGAAGTGGAGGCCATctcgagATGGCCTCCACTTCTGCCTTAtttttg |
|  |  |  | aattcaaaaaTAAGGCAGAAGTGGAGGCCATctcgagATGGCCTCCACTTCTGCCTTA |
| ODF2 | Pbr-31523 | GAGCACATTTGAGGAGACCAA | ccggGAGCACATTTGAGGAGACCAActcgagTTGGTCTCCTCAAATGTGCTCtttttg |
|  |  |  | aattcaaaaaGAGCACATTTGAGGAGACCAActcgagTTGGTCTCCTCAAATGTGCTC |
| RHOBTB3 | Pbr-31524 | GTGGGAAGAATTGGAAGAAGA | ccggGTGGGAAGAATTGGAAGAAGActcgagTCTTCTTCCAATTCTTCCCACtttttg |
|  |  |  | aattcaaaaaGTGGGAAGAATTGGAAGAAGActcgagTCTTCTTCCAATTCTTCCCAC |
| RHOBTB3 | Pbr-31525 | TGGTAATTACATGGAAGCAAA | ccggTGGTAATTACATGGAAGCAAActcgagTTTGCTTCCATGTAATTACCAtttttg |
|  |  |  | aattcaaaaaTGGTAATTACATGGAAGCAAActcgagTTTGCTTCCATGTAATTACCA |
| RHOBTB3 | Pbr-31526 | TCCCGGAAATGTCGTTGCTTA | ccggTCCCGGAAATGTCGTTGCTTActcgagTAAGCAACGACATTTCCGGGAtttttg |
|  |  |  | aattcaaaaaTCCCGGAAATGTCGTTGCTTActcgagTAAGCAACGACATTTCCGGGA |
