# Supplementary material for: Metallopanstimulin-1 (MPS-1) mediates the promotion effect of leptin on colorectal cancer through activation of JNK/c-Jun signaling pathway
Source: Cell Death Dis. 2019 Sep 10;10(9):655. doi: 10.1038/s41419-019-1911-8 (PMC6736844; doi:10.1038/s41419-019-1911-8)
Supplement: Supplementary file 6 — Table S4 [file 41419_2019_1911_MOESM6_ESM.docx]

Table S4 The logFC and *FDR* value of candidates in microarray analysis, and the inhibition rate of cell proliferation on day 5 by shGene

| Gene symbol | logFC | *FDR* | Inhibition rate of cell proliferation at day 5 (%) | *P* value |
| --- | --- | --- | --- | --- |
| LTA4H | 1.205 | 0.002 | 5.763 | 0.083 |
| RRM1 | 1.207 | 0.021 | 11.665 | 0.013 |
| RHOBTB3 | 1.208 | 0.014 | 15.746 | 0.069 |
| MPS-1 | 1.208 | <0.001 | 49.416 | <0.001 |
| IDH2 | 1.208 | 0.039 | 3.903 | 0.293 |
| LAMC1 | 1.208 | 0.019 | 0.909 | 0.920 |
| ODF2 | 1.211 | 0.002 | 20.465 | 0.034 |
| NMD3 | 1.214 | <0.001 | -10.827 | 0.026 |
| PLP2 | 1.215 | 0.001 | 20.782 | 0.004 |
| CDC16 | 1.216 | 0.008 | -4.237 | 0.350 |
