# Supplementary material for: Metallopanstimulin-1 (MPS-1) mediates the promotion effect of leptin on colorectal cancer through activation of JNK/c-Jun signaling pathway
Source: Cell Death Dis. 2019 Sep 10;10(9):655. doi: 10.1038/s41419-019-1911-8 (PMC6736844; doi:10.1038/s41419-019-1911-8)
Supplement: Supplementary file 7 — Supplemetary figure legends [file 41419_2019_1911_MOESM7_ESM.docx]

**Figure S1. The background expression of MPS-1 in 6 CRC cell lines and FHC cells.** The background expression of MPS-1 was detected with qPCR (a) and WB (b) in Caco2, HT29, SW480, HCT116, RKO, DLD-1 and FHC cells. **P*<0.05, ***P*<0.01, ****P*<0.001.

**Figure S2.** **MPS-1 knockdown suppressed proliferation and promoted apoptosis in CRC cells.** (a, b) The knockdown efficiency of MPS-1 in HCT116 cells was evaluated by qPCR (a) and WB (b). (c-f) MPS-1 gene knockdown suppressed cell proliferation (c) and colony formation (d), and induced apoptosis (e) and activity of Caspase 3/7 (f) in RKO cells. Error bars indicate SD of three biological replicates. **P*<0.05, ***P*<0.01, ****P*<0.001.
